# The Mycobacterial Membrane: A Novel Target Space for Anti-tubercular Drugs

**DOI:** 10.3389/fmicb.2018.01627

**Published:** 2018-07-19

**Authors:** Huan Chen, Samuel A. Nyantakyi, Ming Li, Pooja Gopal, Dinah B. Aziz, Tianming Yang, Wilfried Moreira, Martin Gengenbacher, Thomas Dick, Mei L. Go

**Affiliations:** ^1^Department of Pharmacy, Faculty of Science, National University of Singapore, Singapore, Singapore; ^2^Department of Medicine, Yong Loo Lin School of Medicine, National University of Singapore, Singapore, Singapore; ^3^Interdisciplinary Research Group, Singapore-MIT Alliance for Research and Technology, Antimicrobial Resistance Singapore, Singapore, Singapore; ^4^Public Health Research Institute, New Jersey Medical School, Rutgers University, Newark, NJ, United States

**Keywords:** tuberculosis, mycobacterial membrane, drug target, membrane perturbation, cationic amphiphilic indoles

## Abstract

Tuberculosis (TB) poses an enduring threat to global health. Consistently ranked among the top 10 causes of death worldwide since 2000, TB has now exceeded HIV-AIDS in terms of deaths inflicted by a single infectious agent. In spite of recently declining TB incident rates, these decreases have been incremental and fall short of threshold levels required to end the global TB epidemic. As in other infectious diseases, the emergence of resistant organisms poses a major impediment to effective TB control. Resistance in mycobacteria may evolve from genetic mutations in target genes which are transmitted during cell multiplication from mother cells to their progeny. A more insidious form of resistance involves sub-populations of non-growing (“dormant”) mycobacterial persisters. Quiescent and genetically identical to their susceptible counterparts, persisters exhibit non-inheritable drug tolerance. Their prevalence account for the protracted treatment period that is required for the treatment of TB. In order to improve the efficacy of treatment against mycobacterial persisters and drug-resistant organisms, novel antitubercular agents are urgently required. Selective targeting of bacterial membranes has been proposed as a viable therapeutic strategy against infectious diseases. The underpinning rationale is that a functionally intact cell membrane is vital for both replicating and dormant bacteria. Perturbing the membrane would thus disrupt a multitude of embedded targets with lethal pleiotropic consequences, besides limiting the emergence of resistant strains. There is growing interest in exploring small molecules as selective disruptors of the mycobacterial membrane. In this review, we examined the recent literature on different chemotypes with membrane perturbing properties, the mechanisms by which they induce membrane disruption and their potential as anti-TB agents. Cationic amphiphilicity is a signature motif that is required of membrane targeting agents but adherence to this broad physical requirement does not necessarily translate to conformity in terms of biological outcomes. Nor does it ensure selective targeting of mycobacterial membranes. These are unresolved issues that require further investigation.

## Introduction

### Tuberculosis as a global health problem

Tuberculosis (TB) poses an enduring threat to global health. It is currently the world's leading cause of death due to a single infectious agent, surpassing HIV-AIDS and malaria (WHO, 2017). The World Health Organization reported 1.7 million fatalities and 10.4 million new cases of TB in 2016, which is equivalent to a staggering 4700 deaths and 28,500 persons contracting TB per day (WHO, 2017). A major challenge to the eradication of TB is resistance to existing drugs. The past decade has witnessed continued resistance to first line drugs isoniazid and rifampicin (multi-drug resistance, MDR), additional resistance to fluoroquinolones and second-line injectables (extensive drug resistance, XDR), and more recently, cases of programmatically incurable TB where treatment regimens constructed with available drugs completely fail (Dheda et al., 2017; WHO, 2017). A major challenge to TB control is the protracted time required to effect cures with existing therapies. The prevailing consensus points to slow or non-growing bacterial populations (persisters) as the main culprits (Zhang et al., 2012). The factors underpinning the emergence of persisters are multifactorial, stochastic and poorly understood. Persisters are refractory to the host immune system, do not respond to bactericidal antibiotics and serve as reservoirs from which resistant organisms will emerge (Gengenbacher and Kaufmann, 2012). They contribute to the prevalence of latent TB which afflicts nearly 1/3rd of the global population (WHO, 2017). Thus, the key challenge in TB drug discovery is to find structurally and mechanistically novel agents that can eradicate resistant (M/XDR TB) and non-replicating *Mycobacterium tuberculosis (Mtb)*. After decades of relative inactivity, promising drug classes with the potential of treating drug resistant mycobacteria have been discovered (Hoagland et al., 2016). Importantly, many of these agents act on non-canonical targets such as energy production and central metabolism rather than classical macromolecular synthetic pathways (Wellington and Hung, 2018). The emergence of diverse and novel targets should allay concerns of pharmacological redundancies among pipeline candidates in terms of mode of action, cross resistance and side effect profiles (Hoagland et al., 2016).

### Mycobacterial membrane as a putative drug target

There are compelling reasons to support the bacterial membrane as a viable target against resistant and persister organisms (Hurdle et al., 2011). First, all organisms—replicating and non-replicating—are crucially dependent on a functional and physically intact membrane for survival. Second, with nearly a third of cellular proteins concentrated within its confines, the membrane is the site of critical processes such as transport of nutrients and wastes, metabolic energy transduction by respiratory chain enzymes, and cell-to-cell communication in biofilms. Agents that perturb the membrane would disrupt innumerable embedded targets and the associated processes which they support. Third, the lethal pleiotropic consequences of membrane damage would seriously impede the ability of organisms to acquire resistance.

**Figure 1:**
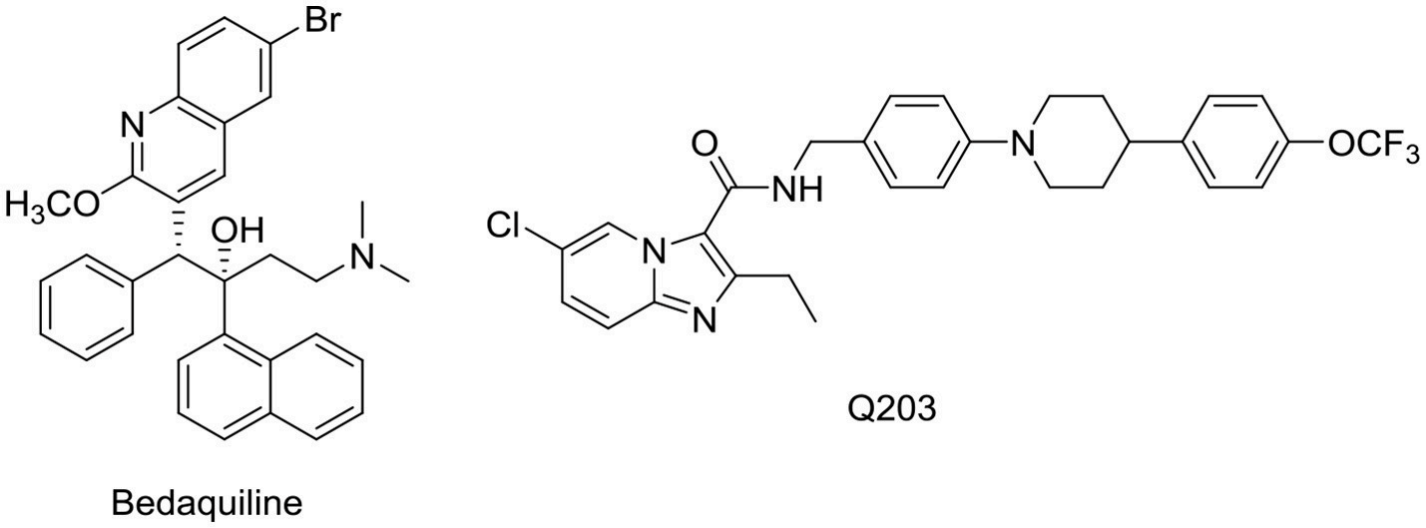
Structures of bedaquiline and Q203. Log P of bedaquiline and Q203 were estimated to be 7.71 and 6.71 respectively. In comparison, the log P of isoniazid, pyrazinamide and ethambutol (standard anti-TB drugs) are −0.64, −1.31, and 0.06 respectively. Log *P*-values were obtained from ChemDraw Professional, Version 15.

Membrane targeting agents may be broadly divided into two classes (Hurdle et al., 2011). In the first class are agents that interact directly with the bacterial membrane bilayer to disrupt its architectural organization and functionality. These agents (cationic amphiphiles) are characterized by their physicochemical properties, notably a threshold level of lipophilicity and a positively charged state that is usually associated with a basic nitrogen moiety. Lipophilicity is required for insertion into the lipid rich matrix of the membrane while the positively charged state will promote selective accumulation within bacterial membranes, which unlike mammalian membranes, are rich in negatively charged phospholipids and polyanionic surface groups (Fischer, 1994; Yeaman and Yount, 2003). The second class of membrane active agents disrupt the function of membrane bound proteins such as enzymes involved in energy production or cell wall biosynthesis. These agents may or may not be cationic amphiphiles. Unlike the first class, their effects on the membrane may be deemed indirect. Examples are the novel anti-TB drug bedaquiline which targets ATP synthase (Andries et al., 2005) and the clinical candidate Q203 which intercepts the mycobacterial cytochrome bc1-aa3 supercomplex (Kalia et al., 2017). Both target critical proteins in the electron transport chain (ETC) (Figure 1). Goldman (Goldman, 2013) noted that phenotypic screening of chemical libraries for new TB leads have had the unexpected outcome of selecting agents that were biased toward membrane targets. This was attributed to the higher lipophilicities of the shortlisted compounds (average log P of 4) which exceeded that of current TB drugs (average log P of 1). In this review, the focus will be on agents that directly target the mycobacterial membrane as this is a relatively unexplored area in the field of TB drug discovery.

## The mycobacterial cell envelope

**Figure 2:**
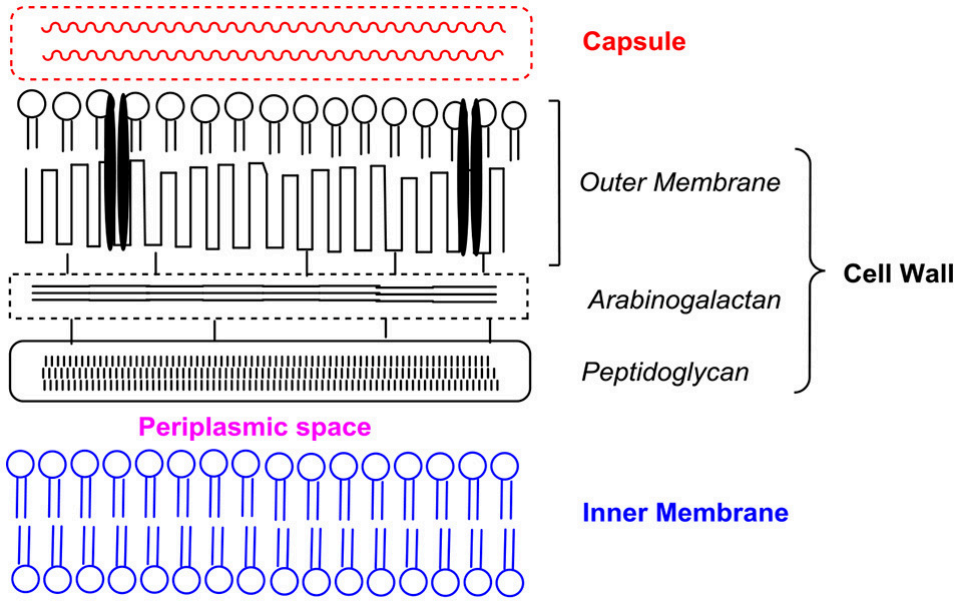
Schematic representation of the different components of the mycobacterial cell envelope based on the description given by Daffé et al. (2017). Presentation is not drawn to scale. Interspersed within the outer membrane are porin channels (depicted as black parallel bars) for the uptake of small hydrophilic molecules.

The mycobacterial cell wall has a distinct architecture that sets it apart from other bacterial cell walls (Brennan, 2003; Hoffmann et al., 2008; Zuber et al., 2008). Mycobacteria are surrounded by a double membrane cell envelope that is exceedingly rich in lipids. No less than 60% of the mycobacterial cell envelope is estimated to be lipids as compared to 20% in the cell envelope of Gram-negative organisms (Brennan and Goren, 1979). Different models have been proposed for the mycobacterial cell envelope (Daffé and Draper, 1998; Marrakchi et al., 2014; Daffé et al., 2017). In one description, the cell envelope is schematically divided into three domains: (i) an outer layer or capsule which is primarily made up of proteins and lesser amounts of carbohydrates and lipids, (ii) a tripartite cell wall comprising an outer membrane (OM) that is covalently linked to an arabinogalactan-peptidoglycan complex, and (iii) an inner membrane (IM) (Daffé et al., 2017). A schematic representation of the cell envelope is given in Figure 2.

Although the double membrane cell envelope is a defining feature of mycobacteria, it was only of late that the OM was disclosed to be a lipid bilayer (Bansal-Mutalik and Nikaido, 2014). The inner leaflet of the OM comprises long chain mycolic acids that are covalently anchored via ester linkages to the underlying arabinogalactan layer. The composition of the outer leaflet of the OM is widely debated, with some sources citing the presence of mycolates, phospholipids and lipoglycans (Chiaradia et al., 2017) while others have argued that it is enriched in diacylglycerols and triacylglycerols (Bansal-Mutalik and Nikaido, 2014). There are also differing opinions about the nature of the IM. One view proposes that it is similar to other bacterial membranes (Bansal-Mutalik and Nikaido, 2014), while another posits that it contains an unusual lipid (diacyl phosphatidylinositol dimannoside) that confers exceptionally low fluidity to the IM (Chiaradia et al., 2017). Taken together, the unique topography and composition of the mycobacterial cell envelope renders it a formidable permeability barrier to external agents and a significant contributor to the general drug resistance phenotype of mycobacteria. How the physical properties of the membrane would influence the physicochemical requirements (lipophilicity, size, polar surface area, pKa) of agents that target mycobacterial membranes is an area that has not been adequately addressed.

## Membrane targeting antimicrobial peptides (AMPs)

Notwithstanding its reputation for impenetrability, the bacterial cell envelope can be breached by antimicrobial peptides (AMPs). AMPs are endogenous polycationic peptides that perform key roles in enhancing innate host defenses against infection. They are generally short chain peptides varying from 7 to 100 amino acids and possess amphipathic/cationic features embedded within their α-helical or β-sheet secondary structures (Zasloff, 2002). Several AMPs are in the clinical phase of development as anti-infective agents (Mahlapuu et al., 2016) but none has been clinically assessed for TB. Nonetheless, several authors have propounded the potential of AMPs as a new treatment strategy against TB (Abedinzadeh et al., 2015; Arranz-Trullén et al., 2017).

The mechanisms by which AMPs interact with bacterial membranes have been widely explored (Silvestro et al., 1997; Park et al., 2000; Yang et al., 2001; Brogden, 2005; Huang and Charron, 2017). Specific steps are involved in peptide mediated membrane disruption. First, the positively charged peptide is electrostatically attracted to the negatively charged bacterial surface (Brogden, 2005). Once anchored to the surface, the peptide transverses the intervening layers (capsule, outer membrane, arabinogalactan-peptidoglycan scaffold) to reach the IM. Translocation may be facilitated by self-promoted uptake in which the peptide destabilizes some component in the cell envelope. The final step involves insertion of the AMP in the IM bilayer and the ensuing loss of membrane permeability. The orientation of the peptide during insertion was noted to be dependent on physical conditions and the composition of the bilayer (Yang et al., 2001). Notably, at low peptide/lipid ratios, the AMP assumes a parallel orientation to the bilayer but at higher ratios, it adopts a perpendicular alignment which is conducive for pore formation. The insertion of the peptide within the membrane bilayer leads to the formation of pores which may be toroidal, barrel stave or carpet-like in nature (Brogden, 2005). Toroidal pores are formed when the AMP bends the lipid bilayer in such a manner that the resulting pore is lined by both the inserted AMP and the lipid head groups. In the barrel stave model, the AMPs aggregate and insert into the membrane bilayer with their hydrophobic regions pointing toward the lipid domains of the bilayer while the hydrophilic regions face inwards to form the pore. Carpet like pores are formed when the AMPs form an extensive layer on the surface of the bilayer and induces disruption in a detergent-like manner (Pouny et al., 1992).

Pore formation compromises the integrity of the membrane by increasing its permeability. This would initiate leakage of ions and metabolites from the cytosol, collapse of the transmembrane potential resulting in membrane dysfunction (impaired osmotic regulation, inhibition of ATP generation via the ETC) and ultimately membrane rupture (Yeaman and Yount, 2003; Brogden, 2005). Intracellular processes such as nucleic acid and protein syntheses may be directly intercepted by AMPs or are the attendant consequences of membrane impairment (Brogden, 2005; Mahlapuu et al., 2016). Thus, the cell killing effects of AMPs are due to multiple and complementary mechanistic pathways.

Considerable variation in this canonical model has been noted for specific AMPs. For example, penetratin did not induce pore formation in bacterial membranes in spite of its cationic amphipathic character (Huang and Charron, 2017) while buforin II penetrated the cytoplasmic membrane and accumulated within the cytosol, without incurring significant damage to the membrane (Park et al., 2000). Cecropin A displayed a concentration-dependent effect, dissipating transmembrane electrochemical ion gradients in lipid vesicles at low concentrations and permeabilizing lipid vesicles at higher concentrations (Silvestro et al., 1997).

Ubiquitin-derived peptides have been reported to contribute to the mycobactericidal activity of lysosomes (Alonso et al., 2007). Investigations on a synthetic ubiquitin peptide (Ub2) revealed the involvement of other interactions with the mycobacteria (Purdy et al., 2009). In keeping with its cationic amphiphilic character, Ub2 disrupted the membrane integrity of mycobacteria. Interestingly, *M. smegmatis* mutants which were resistant to Ub2 displayed reduced mycobacterial cell wall permeability which would in turn hinder access of the peptide to the IM. Tellingly, some of these mutants also lacked the major porin MspA but entry of Ub2 into the bacilli was not impeded by the loss of MspA channel activity. Rather absence of the porin diminished permeability of the outer membrane, an observation that has been noted by others (Stephan et al., 2004). These changes in membrane permeability also reduced the ability of macrophages to eliminate Ub2-resistant mutants. Thus, the decrease in outer membrane permeability was pivotal to the emergence of the Ub2-resistance phenotype of *mspA* mutants.

Despite several appealing properties (rapid killing, low immunogenicity, uptake by macrophages, immunomodulatory properties) which render AMPs highly attractive as antimycobacterial candidates, their clinical advancement has been hampered by stability and pharmacokinetic issues arising from their peptidic character (Arranz-Trullén et al., 2017). Non-peptidic membrane targeting small molecules would arguably overcome these limitations and several such entities have been investigated against Gram-positive and Gram-negative bacteria with promising outcomes (Vooturi et al., 2009, 2011; Eun et al., 2012; Zou et al., 2013; Hurley et al., 2015; Faulkner et al., 2016; Koh et al., 2016a; Wang et al., 2016; Shuimu et al., 2017; Su et al., 2017). In comparison, the concept of damaging the structure of mycobacterial membrane bilayer as a therapeutic strategy has received less attention. In the following paragraphs, we review these agents with regard to their SARs and mechanistic effects on the mycobacterial membranes.

## Membrane targeting xanthones

**Figure 3:**
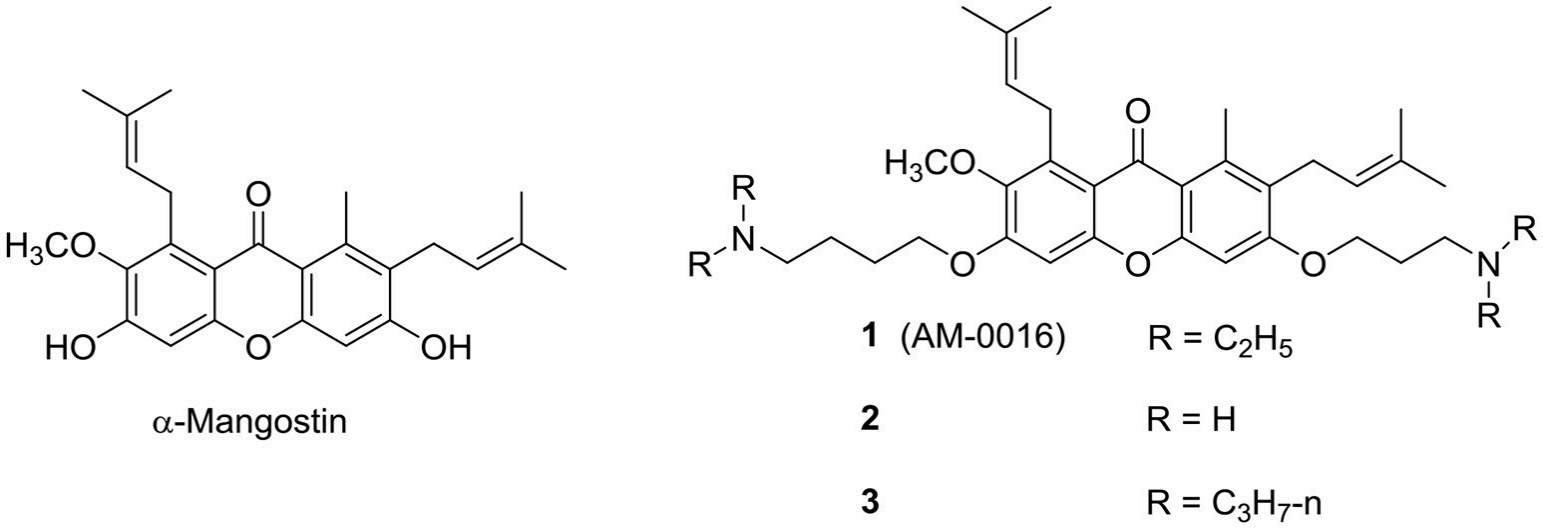
α-Mangostin-derived xanthones with antimycobacterial activity.

α-Mangostin, a xanthone isolated from the tropical plant *Garcinia mangostana*, was reported to possess potent antibacterial activity against Gram-positive organisms, including methicillin resistant *Staphylococcus aureus* (MRSA) (Koh et al., 2013). Its mode of action was attributed to disruption of the bacterial membrane, but a lack of selective toxicity precluded it from further clinical development. Nonetheless the xanthone ring was deemed a promising scaffold for structure optimization and the approach of inserting polar cationic residues onto the lipophilic xanthone successfully led to potent analogs **1** (AM-0016) and **2** (Zou et al., 2013; Koh et al., 2016a) (Figure 3). These compounds had low to submicromolar minimum inhibitory concentrations (MICs) against MRSA, rapid bactericidal activities, excellent cytotoxic selectivity and efficacy in a mouse model of corneal infection caused by MRSA and *S. aureus*. Both **1** and **2** induced permeabilization of *S. aureus* (increase in fluorescence of SYTOX Green) but only **1** (AM-0016) dissipated the membrane potential when investigated with the cytoplasmic membrane potential-sensitive dye DiSC3-5.

AM-0016 was subsequently reported to be bactericidal against *Mtb* (Mukherjee et al., 2016). It displayed a rapid time-kill profile, activity against hypoxic non-growing *M. bovis* BCG cultures and a low spontaneous resistance frequency. Similar to its actions on *S. aureus*, AM-0016 induced a rapid collapse of the membrane potential and induced membrane permeabilization in *M. bov*is BCG. Both events were incurred before significant loss in cell viability and overt damage to the cell envelope. The cidal effects of AM-0016 may be due to direct effects on the cell wall components or from downstream changes consequent to the collapse of the membrane potential. Increasing the lipophilicity of AM-0016 by homologation of the terminal diethylamino side chains improved the overall mycobactericidal profile (Koh et al., 2016b). One such analog (**3**) exhibited favorable pharmacokinetics in mice. Like AM-0016, the antimycobacterial activity of **3** involved a loss in membrane integrity.

## Boromycin targets the mycobacterial membrane ion gradient

**Figure 4:**
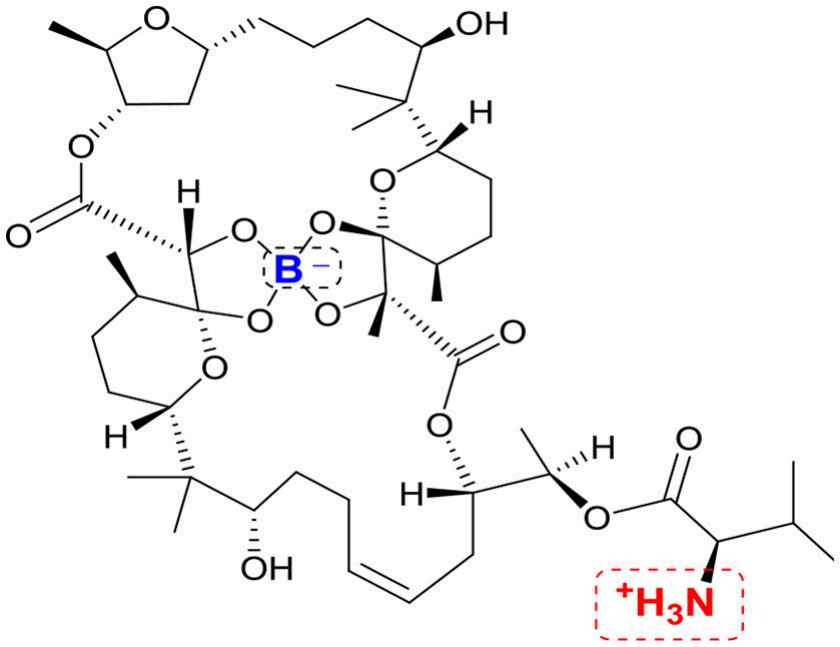
Structure of boromycin. Positively charged amino group and negatively charged boron atom are highlighted in red and blue respectively.

Boromycin is a boron-containing polyether-macrolide antibiotic isolated from *Streptomyces antibioticus*. It is bactericidal against Gram-positive organisms where it functions as an ionophore to abolish the potassium ion gradient across the bacterial membrane (Pache and Zähner, 1969). Boromycin is not effective against Gram-negative bacteria, presumably due to the presence of an outer membrane which would block its access to the cytoplasmic membrane. (Hütter et al., 1967; Pache and Zähner, 1969). Thus, it was with some surprise that boromycin was found to be bactericidal against mycobacteria which also possess a double membrane cell envelope. Boromycin induced collapse of the mycobacterial transmembrane potential which led to leakage of cellular contents (Moreira et al., 2016). The compound potently inhibited mycobacterial growth (MIC_90_ 200 nM, *Mtb*), was active against hypoxic non-replicating persisters, and demonstrated a favorable selective toxicity against mycobacteria (selective index > 300). It had a low spontaneous mutation frequency (<10^−9^/CFU) which was in keeping with its membrane targeting effects. The exogenous addition of potassium abolished the antimycobacterial activity of boromycin. Structurally, boromycin is not a typical cationic amphiphile. Although it has a positively charged amino group, this is neutralized by the negatively charged boron that is embedded within the macrocyclic ring (Figure 4). Boromycin is also rich in polar hydrogen bonding groups and its solubility in methanol and ethanol suggests that it is not overly lipophilic. Thus, its ability to transverse and perturb the mycobacterial cell envelope is puzzling and reinforces the notion that molecules are attracted and attached to the cell envelope by varied means, most of which are not well understood.

## Membrane targeting cationic amphiphilic indolyl mannich bases

**Figure 5:**
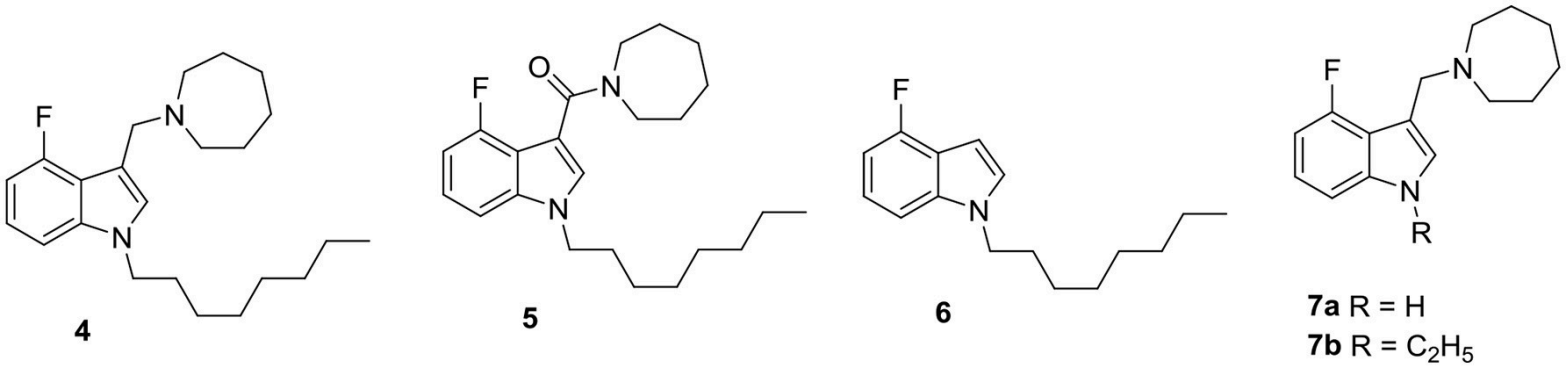
The cationic amphiphilic indolyl Mannich base **4** is a membrane targeting mycobactericidal agent. Cationic amphiphilicity is absent in **5, 6, 7a**, and **7b** which are deconstructed analogs of **4**. They have poor antimycobacterial activities.

Our interest in membrane targeting antimycobacterials came about when a routine screen of indolyl Mannich bases identified several members with low micromolar MICs against *Mtb* (Yang et al., 2017). The presence of the lipophilic n-octyl side chain and positively charged basic nitrogen atoms in these entities qualified them as cationic amphiphiles and prompted the question as to whether this motif was causal to antimycobacterial activity. To this end, we deconstructed one of the potent compounds (**4**) to yield analogs that retained either the lipophilic feature or the basic center, but not both. Removal of the basic center gave **5** and **6** which retained the n-octyl side chain whereas in **7a** and **7b**, the basic nitrogen atoms were preserved but not the non-polar side chain (Figure 5). As anticipated, the loss of either feature abolished antimycobacterial activity. To determine if membrane perturbation contributed to the activity of **4**, several approaches were investigated. First, negatively charged dimyristoylphosphatidyl-glycerol (DMPG) vesicles were prepared as a surrogate of the anionic bacterial membrane (Pinheiro et al., 2013). The melting profiles of these vesicles were then monitored by differential scanning calorimetry. Compound-induced perturbation of the vesicular bilayer would result in the broadening of the melting endotherm of the phospholipid, a downward shift in its phase transition temperature Tm and a reduction in the calorimetric enthalpy (ΔH) of transition (Jain and Wu, 1977; Seydel, 2002). These changes were duly observed for vesicles containing **4** but were strikingly diminished in those containing **5-7**. Furthermore, the sharp loss in the ΔH of the melting endotherm observed in vesicles containing **4** pointed to weakened interactions within the DMPG bilayer which suggested that **4** was embedded within the hydrophobic core of the lipid bilayer.

As reviewed earlier for membrane targeting antibacterial agents, disruption of the membrane would result in permeabilization and depolarization. We found that **4** depolarized the mycobacterial membrane, as evidenced from the decrease in the red/green fluorescence ratio of the membrane probe DiOC_2_ after exposure to *M. bovis* BCG for 48 h. It also increased the cytoplasmic membrane uptake of the fluorescence probe propidium iodide (PI) in *M. bovis* BCG within 24 h (Yang et al., 2017). To further confirm the membrane disruptive effects of **4**, we examined its effects on the mycobacterial *iniBAC* gene cluster. The transcriptional competency of this cluster is induced by agents that increase cell envelope stress (Alland et al., 2000). We found that **4** induced *ini*BAC promoter activity in recombinant *M. bovis*-p*iniBAC*-RFP cultures. When tested at its MIC_50_, **4** increased activity by 10-fold compared to untreated controls. Interestingly, *M. bovis* BCG mutants that were resistant to **4** could not be isolated even after prolonged exposure (8 weeks). The low mutation frequency (<10^−9^/CFU) together with evidence from the biophysical (disruption of DMPG vesicles), biochemical (membrane permeabilization) and genetic (induction of p*iniBAC* operon) experiments point to a causal link between the mycobactericidal activity of **4** and its membrane corrupting effects. Importantly, the perturbative effects of **4** were selective for bacterial membranes and did not extend to mammalian membranes. The hemolytic activity of **4** on human red blood cells (50% hemolysis observed at 35 μM relative to Triton X-100) and cytotoxicity against mammalian African Green Monkey Vero cells (IC_50_ 29 μM) were observed at concentrations that were 10 times higher than its MIC. In spite of its lipophilic character (clogP 5.97), **4** was reasonably soluble (kinetic solubility of 37 μM determined after 24 h, 25°C, pH 7.4). It was also found to have a long *in vitro* half-life (>100 min, rat liver microsomes) (Yang et al., 2017). Synthetic attempts to optimize the antimycobacterial activity of **4** are ongoing.

## Membrane targeting cationic amphiphilic indolylalkyltriphenylphosphonium analogs

**Figure 6:**
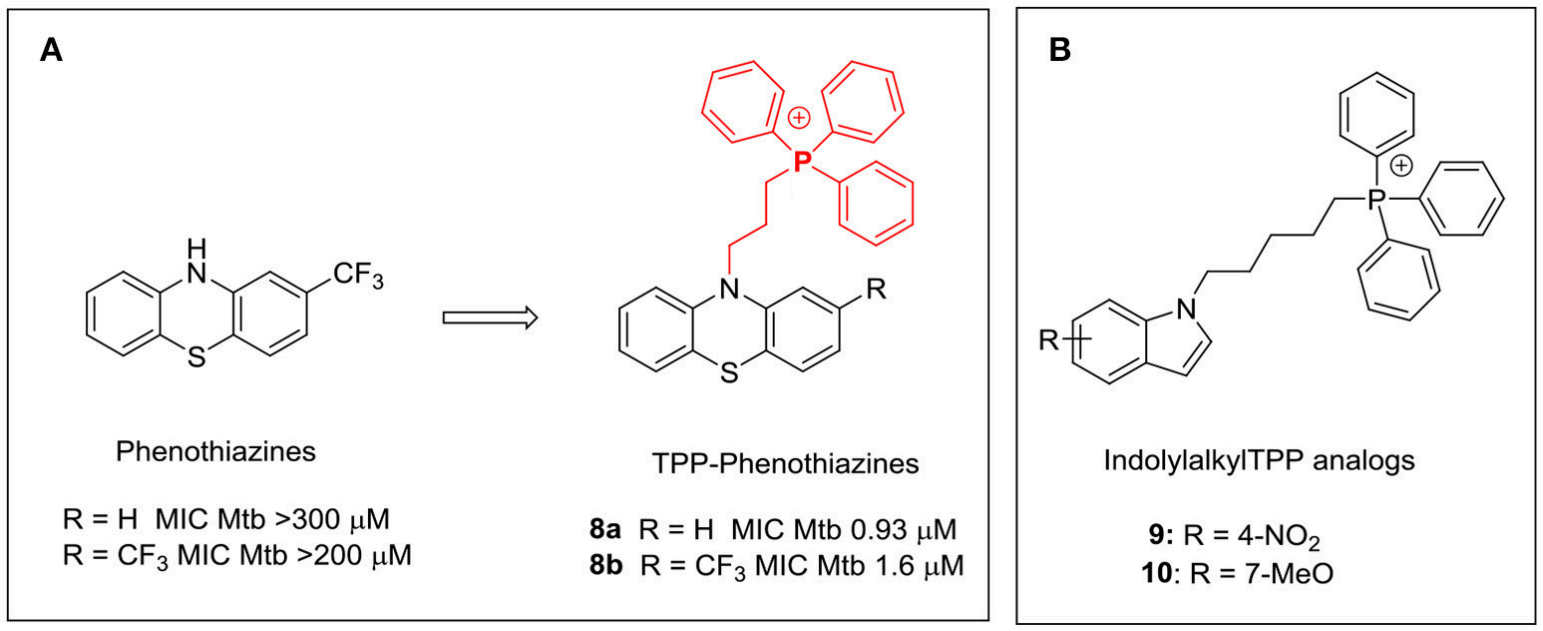
**(A)** Attachment of a triphenylphosphonium side chain increased the antimycobacterial activity of phenothiazines **8a** and **8b** (Dunn et al., 2014); **(B)** Antimycobacterial TPP-indoles **9** and **10** with membrane targeting properties (Li et al., 2017).

The triphenylphosphonium (TPP) cation is widely employed as a membrane targeting motif (Ross et al., 2008; Madak and Neamati, 2015). The lipophilicity of the TPP cation coupled with its large ionic radius and extensive delocalization of the positive charge favor accumulation within cellular compartments with large negative membrane potentials such as the mitochondria (Δψ: −170 mV) and most bacteria (Δψ: −135 to −180 mV) (Krulwich et al., 2011; Madak and Neamati, 2015). TPP conjugates of several antioxidants and anticancer agents that specifically accumulate in the mitochondria showed significant gains in activity (Yousif et al., 2009; Madak and Neamati, 2015). The phenothiazine thioridazine has been shown to be effective against XDR-TB (Amaral et al., 2010). Dunn and coworkers showed that attaching a TPP moiety to the phenothiazine scaffold greatly enhanced activity, presumably due to localization of the phenothiazine-TPP conjugates (**8a, 8b**) within the vicinity of NDH-2, the membrane bound oxidoreductase which is widely acknowledged as the target of antimycobacterial phenothiazines (Figure 6A; Dunn et al., 2014). Adoption of this approach may enhance the therapeutic potential of this class of compounds against TB.

In an effort to explore the structural diversity of indole-based cationic amphiphiles with membrane targeting antimycobacterial activities, we prepared a series of indolylalkyltriphenylphosphonium analogs (TPP-indoles, Li et al., 2017). Unlike the indole based Mannich bases exemplified by **4** where the positive charge and lipophilicity were located separately in the same molecule, both features were embedded within the TPP core in this series. Several analogs (**9, 10**) with low micromolar MICs were identified (Figure 6B). These compounds were equipotent in terms of their bactericidal activities with MIC_90Mtb_ and MBC_99.9BCG_ of 6 μM. Several key observations were made with regard to SAR. First, loss of activity was observed when the TPP moiety was omitted. Second, analogs were more potent but less selective when the methylene linker between TPP and the indole ring was extended from 3 to 5 carbon atoms. Third, there was considerable tolerance for substitution on the indole ring. Lastly, omission of the indole ring from **9** and **10** reduced activity but a rebound was noted when sufficiently lipophilic entities were introduced in place of the ring. Thus n-decyl TPP and phenylhexyl TPP cations had MICs that were comparable to **9** and **10**. Unusually, both **9** and **10** did not increase membrane permeability in *M. bovis* BCG. Neither did they increase cell envelope stress by inducing p*iniBAC* activity nor change the melting profiles of DMPG vesicles as observed for **4**. On the other hand, **9** and **10** caused rapid and sustained depolarization of *M*. *bovis* BCG which led to disrupted electron transfer in the ETC and abnormal cell division in *M. bovis* BCG. Thus, the TPP-indoles **9** and **10** have the surprising effect of dissipating the transmembrane potential without concurrent loss in membrane permeability. There was however precedent to the decoupling of these closely related phenomena (Okano et al., 2017). This was observed in a highly potent vancomycin analog that was modified by inserting a positively charged quaternary trimethylammonium moiety at a peripheral C-terminal with the intent of inducing membrane permeability. Only membrane permeabilization and not depolarization was observed in cultures of vancomycin-resistant *Enterococcus faecium* (VRE, *vanA* gene) treated with the vancomycin analog. The authors posited that the analog may be involved in “specific interactions within the bacterial cell wall” which led to the loss in membrane permeability (Okano et al., 2017).

**Figure 7:**
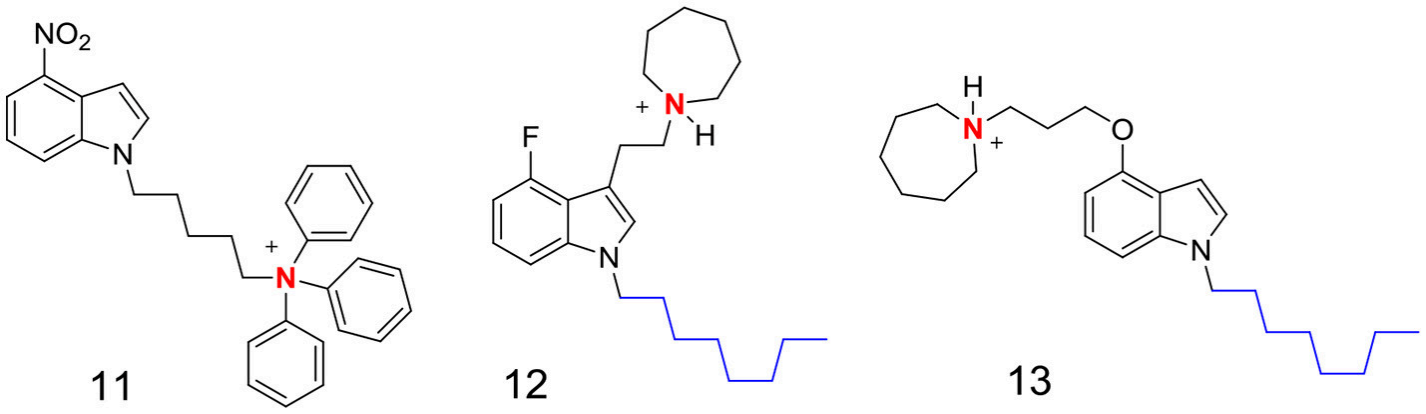
Proposed indole-based cationic amphiphilic scaffolds. In **11**, the quarternary triphenyl ammonium moiety bears the positive charge (in red) and the lipophilic phenyl rings. In **12** and **13**, the n-octyl side chain is lipophilic (blue) and the basic nitrogen (red) is protonated (positively charged) at pH 7.4 and lower.

The contrasting profiles of the indolyl Mannich bases (exemplified by **4**) and the TPP-indoles (**9, 10**) prompted us to reassess the membrane disrupting propensity of the cationic amphiphilic motif. While it is apparent that the presence of the motif directs the molecule to the membrane, it does not determine how the membrane would ultimately be perturbed. Thus, the TPP-indoles targeted a critical membrane-based process (ETC) whereas the indolyl Mannich bases induced general membrane permeabilization. Perhaps, these differences are due to the distinct topography of the cationic amphiphilic motif in each scaffold. As mentioned earlier, the TPP moiety served as both a center of positive charge and lipophilicity in the TPP-indoles whereas these features were found in different parts of the indolyl Mannich base. It might be worthwhile exploring other indole-based cationic amphiphilic scaffolds to determine if the orientation of these features is truly important. Some possible scaffolds are proposed in Figure 7. Charge and lipophilicity are not segregated in **11**, similar to the TPP indoles whereas **12** and **13** are mimics of **4**, with the important difference that they are not Mannich bases (Figure 7). Support for the hypothesis would require **11** to perturb mycobacterial membranes in the same way as the TPP indoles, and for **12** and **13** to behave like the indolyl Mannich base **4**.

## Verapamil disrupts membrane energetics of mycobacteria

**Figure 8:**
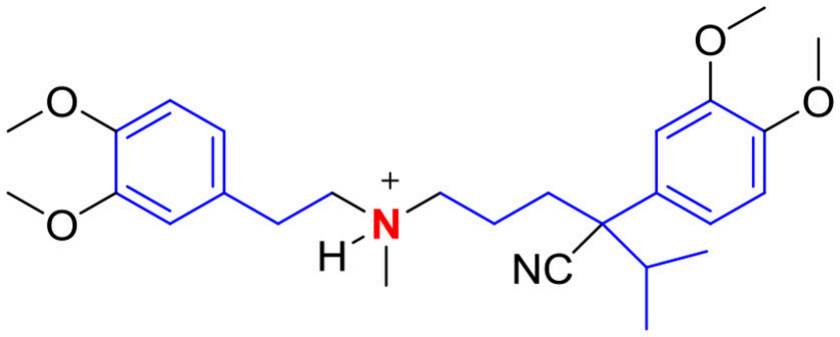
Verapamil is a cationic amphiphile with a basic tertiary amino group (red) and lipophilic residues (blue).

The p-glycoprotein efflux pump inhibitor verapamil has been reported to restore the activity of several first line anti-TB drugs against drug-resistant isolates (Pasca et al., 2004; Srivastava et al., 2010; Machado et al., 2012; Sun et al., 2014) and to potentiate the *in vitro* activity of newer anti-TB agents bedaquiline and clofazimine on *Mtb* cultures (Gupta et al., 2014). These effects are widely attributed to the suppression of bacterial efflux pumps by verapamil which would diminish expulsion of the anti-TB agent from the mycobacteria. Consequently, higher concentrations of the agent would accumulate within the organism, leading to greater cidality. Interestingly, this notion has not been experimentally verified. In an attempt to elucidate the mechanistic basis underlying verapamil-mediated potentiation of anti-TB drugs, Chen and co-workers discovered an unexpected pivotal role for the mycobacterial membrane (Chen et al., 2018). Their investigations revealed that verapamil (at 128 μM, equivalent to ¼ MIC) potentiated the growth inhibitory activities of bedaquiline and clofazimine on susceptible and multi-drug resistant strains of *Mtb*. The potentiation was synergistic but contrary to expectations, was not accompanied by enhanced accumulation of either drug in mycobacteria. In fact, the intracellular concentrations of bedaquiline or clofazimine in cultures that were pretreated with verapamil were not significantly different from cultures that were not pretreated. The authors then showed that verapamil caused a rapid and concentration dependent collapse of the mycobacterial membrane potential and that the loss in membrane potential preceded bacterial cell death. Membrane depolarization by verapamil was consistent with its ability to synergize with bedaquiline and clofazimine whose modes of action (disruption of oxidative phosphorylation) required intact and functional membranes. Tellingly verapamil did not synergize with rifampicin which is not membrane targeting and does not intercept oxidative phosphorylation. The ability of verapamil to target the mycobacterial membrane is in keeping with its cationic amphiphilic nature (Figure 8). As a lipophilic base that is extensively protonated at pH ≤ 7.4, verapamil would have a strong affinity for lipid bilayers, as observed by others (Pohl et al., 1998). In the same way, accumulation of verapamil within the mycobacterial membrane would advantageously position it for disruption of the membrane potential. That verapamil directly impacted membrane energetics was subsequently confirmed in experiments with inverted membrane vesicles prepared from *M. bovis* BCG (Chen et al., 2018). The results pointed to preferential interception of the ΔpH component of the proton motive force. Interestingly, verapamil did not permeabilize mycobacterial cultures although it did induce the transcriptional activity of genes (*sig*E, *clg*R) corresponding to reporters of membrane stress. Thus, notwithstanding the stress imposed by verapamil on mycobacterial membranes, it also induced processes that would paradoxically preserve membrane function. In all, this report highlighted the previously unrecognized role of verapamil as a depolarizer of mycobacterial membranes and by extension, the relevance of mycobacterial membranes as a drug target.

## Challenges to agents targeting mycobacterial membranes

The selective targeting of mycobacterial membranes is associated with unique challenges not encountered with other discovery approaches. Two main hurdles limit the potential of membrane targeting agents for TB. The first relates to a knowledge gap as to how early hit molecules could be structurally modified to achieve greater antimycobacterial potency and selectivity. This problem is not peculiar to mycobacteria but may be more acute in view of the complexity of the mycobacterial cell envelope as compared to other bacteria. As mentioned earlier, attraction for the membrane is driven by the physicochemical nature of the entity, namely cationic amphiphilicity. This is a general requirement fulfilled by structurally diverse chemotypes but our own experience with the indole analogs (**4, 9, 10**) have shown that adherence to this broad physical requirement does not necessarily translate to conformity in terms of biological outcomes. Thus, we found that the TPP-indoles (**9, 10**) depolarized mycobacterial membranes without affecting membrane integrity. The same profile was observed for verapamil. In contrast, both permeabilization and depolarization were noted for the xanthone AM0016 (**1**) and the indolyl Mannich base **4**. Clearly, serious gaps exist in our understanding of structure-membrane disruption relationships.

The second hurdle concerns the selective activity of membrane targeting agents. Being lipophilic, these agents would undoubtedly partition into mammalian membranes and cause toxic off-target effects. A case in point is oritavancin, an antimicrobial glycopeptide with membrane disrupting properties that induced mixed-lipid storage disorders in macrophages and fibroblasts (Van Bambeke et al., 2005). Goldman cautioned that while AMPs may have a preference for bacterial over mammalian membranes, this may not be true for membrane targeting small molecules (Goldman, 2013). Although the Global Health Innovative Technology (GHIT) Fund requires TB lead compounds to possess a 10-fold selectivity in killing pathogens as opposed to mammalian cells (Katsuno et al., 2015), most investigators impose a higher cytotoxicity threshold for their leads. Cell based cytotoxicity assays should be supplemented by the red blood cell hemolysis assay which provides a simple and effective means of eliminating molecules that damage mammalian membranes. Ultimately, *in vivo* acute and chronic toxicity studies would be necessary for the most promising leads.

Challenges notwithstanding, selective targeting of mycobacterial membranes offers an untapped opportunity to address the problems associated with current TB drugs. As more attention is drawn to its therapeutic potential, a better understanding of the science underlying the disruptive processes will emerge and this will ultimately lead to the development of more potent and safer drugs against active and latent tuberculous infections.

## Author contributions

TD and MLG proposed the concept. HC, SN, ML, PG, DA, TY, WM, and MG planned and conducted the experiments in our reported work. TD, MG, PG, WM, SN, ML, and MLG contributed to the writing and editing of the manuscript.

### Conflict of interest statement

The authors declare that the research was conducted in the absence of any commercial or financial relationships that could be construed as a potential conflict of interest.
